# Worried to Death

**Published:** 1882-01

**Authors:** Ben. H. Pratt


### THE BISTOURY.

Vol. ~~XVIII.~~ 17 ELMIRA, N. Y., JANUARY, 1882. No. 4.

### Contributions.

### WORRIED TO DEATH.

BEN H. PRATT.

There is not much doubt that people have actually died from disease—purely physical disease. Some—not many—live to be old enough to die from sheer age. People generally kill themselves, however. If people kill themselves, then are they suicides. That goes without saying. We are accustomed to rather stand in awe of suicide, when we know it to be such. If a man cut his throat, and in doing so do not make a bungle of it; if he do a clean, thorough, artistically surgical job, cutting off his jugular vein as well as his trachea; we gather around him with a species of combined admiration of the fellow's spunk, and respect for his skill and anatomical achievement. If he blow the top of his head off with a big calibered, competent, well aimed pistol or gun shot, we assemble at his residence and sort of lionize his memory. Even if a man shows keen wit and chemical knowledge enough to select and procure and administer to himself a neat, effectual and not too irritating a drug, and ends his career quietly and without any previous brag and bluster, we drop in on the remains, and look him over with profound regard for his pluck, and his willingness to take his chances on the next existence. Probably the reason we do these things is because we have somehow or other got it into our heads that those who kill themselves are exceptional people. They seem to us to be out of the common run of mankind, so to speak; and whatever is out of the common run is admirable in our eyes.

It's a mistake. If we would only stop and think a little, we'd see how very much off we are in drawing such a conclusion. Suicides are not rare. Nine-tenths of our race—that is, the civilized portion of it—die suicides. I grant you, they don't all cut their throats, or blow out their brains, or swallow poison, or rip themselves open, or throw themselves over precipices and water falls and off from bridges and docks, or hang themselves to barn-beams and bed-posts, or take other varied and violent measures to get rid of living; but they commit suicide all the same, though they don't get the credit for it. Perhaps they don't deserve credit for their acts, because it is very possible that they do not realize that they are committing suicide; though how they can avoid knowing it is a mystery.

You don't mean to say that you don't understand how nine-tenths of the civilized world are committing suicide, and you right among them, and watching them do it! Surely you doctors ought to understand it. You don't talk it, though. You get the credit of doing most of the killing yourselves, but you don't deserve the reputation.

Human animals are the most inconsistent creatures on the face of the earth. Now look at them! Here they are, nearly every one of them ailing somewhere; complaining of something; fearfully worked up over a fear that something awful is the matter with them; calling on the doctors to save them; dosing themselves with nostrums of all sorts, prescribed and not prescribed; eternally swallowing something, doing something, rubbing with something, applying something, going somewhere; all to help their alleged infirmity, or malady, or trouble, or indisposition, or invalidity, or bad feelings, or strange sensations, or something. They doctor and dose and rub and swab and bathe and exercise and annoint and inhale and travel and turn their worldly affairs upside down and topsy-turvy to get rid of a real or a fancied ailment, and then blame the doctors because they don't get well or can't get well, and then say that the doctors are killing them.

Now, it isn't the doctor that's killing them; nor is it the dosing or rubbing or anointing or plastering or poulticing or running about. It is the worrying. Now we're on it. Nine-tenths of mankind—civilized folks, so called—die of worriment. They kill themselves worrying over the fear that they are dying by inches. What's worse, half of them know that it's the worry, and not the effect of the worry that's killing them. The rest ought to know it, if not from what they are told, from what they see. People with lots of power of observation in matters that don't concern them in the least, become perfect ninnies when they themselves become the objects of their observations- They know how other people ought to do, in the light of never varying experience, but won't see, and can't be made to see how they ought themselves to act. They see people going right along worrying themselves into their graves, and yet they'll keep on with the same fatal worry, entertaining the singular hallucination that what kills others will save them. "All men think all men mortal but themselves." Yes, worrying kills nine-tenths of us, and we keep on worrying, and keep on killing ourselves—committing suicide.

Worrying is such a one-sided evil that one would think people would avoid it. What good has ever come of worrying? If it brought us an occasional boon it wouldn't be so surprising that we courted it; but it doesn't. Fretting and stewing and wishing that things thus and so were some other way, don't ever make the thus and so otherwise. It makes them ten times more thus and so and ten times less otherwise. But we keep right on fretting, and inviting ourselves into misery deeper and deeper, and by and by we die, killed by fretting and stewing and worrying. There is nothing will make a man sick quicker or sick more thoroughly than imagining himself sick. Imagination is the eager handmaid of whatever enters the mind. If we have a healthy idea, imagination makes it healthier—strengthens it, and develops it. If we have a sickly, silly notion, imagination makes it sicklier and sillier. Imagination isn't particular what kind of seed you sow in the mind. It will enrich the mind for the bad, the weak, the silly, just as thoroughly as it will for the good, the strong, the wise. Whatever you take into and cherish within the mind will be fructified by the imagination, whether it be moral figs or thistles, grapes or thorns, fish or serpents. Imagination, working on good mental seed, produces wholesome thought, genius, benevolence, enterprise, affection, love, all the qualities that elevate the race; working on bad seed, it develops moroseness, despondency, hate, melevolence, misanthropy, hypochondria, disease, death.

I read only the other day of a doctor having been called to minister to a woman who imagagined she had swallowed a whole set of false teeth during her sleep. She conceived the idea that the teeth were in her stomach, and she couldn't be persuaded otherwise. She couldn't tell when nor how she swallowed them. It made no difference to her that a full set of fourteen teeth, plate and all, couldn't be crowded down a human throat. Comparative measurements were of no account. If the teeth had been as big as a stove lid and her throat as small as a pipe stem, 'twould have made no difference. She knew that she had swallowed them without choking, and their sharp edges were poking into her stomach lining, and she was getting sicker and sicker and knew she'd die inside of an hour. And it is likely that she would, hadn't the doctor searched the bed and found the teeth between the pillow and pillow-case, just where she had put them before going to sleep. She recovered immediately upon being convinced she had been a fool.

I recall an impressive incident of my boyhood. A member of my own family was in the habit of tickling her ears with a pin head. It was many years ago, when round-headed pins were made, and sometimes, as you know, the heads used to come off. One evening she was titillating her ear with one of these pins, and upon removing and examining it she discovered that the pin was headless. She knew the head had dropped into her ear—was sure it had gone into her brain—would there inflame that organ. She was confident she would soon be crazy—she knew it would kill her. The doctor was sent for, the patient became weaker and weaker, took her bed, grew worse rapidly and became wild and incoherent in her talk by reason of the action of the foreign substance in her brain. She knew enough of anatomy to realize that it couldn't reach her brain, but sense doesn't amount to shucks when imagination has control. She knew she was dying, but before she succumbed, the pin head was found on the carpet near where she had sat to pick her ear, and she was just alive enough to comprehend the situation. In ten minutes she was well, but the neighborhood never forgave her the scare she gave them.

Imagination makes fools of men and women, and I cite the above incidents in proof of its supremecy over sense and even over wisdom and vast learning. It is the greatest blessing and the foulest curse of mankind, but whether it becomes for you and me a blessing or a curse, depends upon what we put within our minds for it to work upon. Put therein pure and noble thought-seed, and imagination will cultivate and develop the maturing growth, and the fruition will be healthful, ennobling, manlike. Drop therein but a germ of ill, and there comes forth under imagination's incubation, a brood of worse than demons to rack and wreck and kill.

Worriment is not an indication of mental strength, or mental activity, or mental sensitiveness. We are too apt to call a man a dull fellow because he doesn't worry and fret and get himself into a stew over the little or big provocations that force themselves into the life of every one, to a greater or less extent. We say of the stolid, philosophical, easy going, phlegmatic fellow, "O, he's too dull, or too stupid to be endured; he hasn't any mind; he can't have any soul, to let the ills of life fall upon and around him and not be driven into a frenzy over them; he lacks some essential quality of mind; he isn't altogether normal." Not so. He is simply one who has sown such thought-seed, such germs of sentience in his mind, that imagination, the cultivator and fructifier, develops a different sort of mental product from that which is borne by the carelessly and indiscriminately planted mind. As few are careful and many are indifferent as to what they stow away in their minds, so few are the controllers of their own mental products and many are the slaves thereof.

Men and women are worrying themselves to death every day, and the number is so great as to make the man or woman who doesn't so worry unto death the exception. Fretting grows with fearful rapidity upon those who yield to it. When the fretful soul has nothing real to worry over, it conceives something. It must have material to work upon. Worrying becomes chronic, and like the thrist for drink, or the longing for the sensational, or the craving for social excitement, will always find something whereon to exercise its restless mandibles. Men and women worry and fret with marvelous universality. One would think that the rich, having all they can get that is good for them to have, would cease to worry. But they don't. The rich and poor, learned and ignorant, cultivated and uncouth, worry with equal intensity. The poor worry because they are poor. Those in comfortable circumstances worry lest something may happen to make them poor. The rich worry over the added cares of wealth and with their strivings to be richer. The ignorant worry because they do not know as much as their neighbors, and the educated worry because they know how little they know of what they might know. Everybody compares his own lot with that of some one above him in station, and worries because somebody whom he knows has something that he hasn't. Men fret and worry because their business doesn't run along with absolute smoothness, and because there are continually arising unexpected episodes to prevent the easy accomplishment of their previously laid plans. They fret and fume over petty annoyances in the household, just as if their household was different from that of any other household. Women worry and fret over their cares and duties, and the less care and responsibility they have, the harder they fret. "*Experiencia docet!*" Bosh! It doesn't do anything of the kind. The more we see of the ill effects of worrying, the more we worry. Experience doesn't teach. Nothing will teach but a right strong up and down determination to take things as they come, and make the best of them. Life is too short to waste in worrying, and we shorten it through our worrying by many years. We worry at possible misfortunes, and bring them into our mental view long before they reach us, and when they do come, if they come, we worry because they are not half as big as we imagined them to be, and we've had all our worry for nought. This makes us worry more. If men and women could only summon up philosophy enough to determine not to bother over ills until they actually get to them, there would be mighty few of those ills that would live to get to them. It is wonderful to what a size anticipation swells a distant event, whether it be a pleasurable or a dreaded one. As there is more enjoyment in anticipating something we expect to derive pleasure from than there is in the actual experience of that something—and we all know that is true—so the dread of an anticipated evil is ten fold greater than the evil itself when it does come. We are often chagrined to find what we have dreaded turn out to be of no consequence at all; and we have all of us, many a time, called ourselves fools for allowing ourselves to make ourselves miserable over something that has actually turned out to be a pleasure instead of a pain. Whoever heard of anybody's gaining anything by worrying? Who doesn't know of somebody who is wholly unfitting himself or herself for the most ordinary duties of life by continual worrying Who of us are there but can truthfully confess that we worry over hundreds of ills that never come, and have sacrificed from a third to nine-tenths of the enjoyments we might have had but for our fretting. The human mind, in its natural state, is a joy giver and a joy receiver. It's natural for people to be happy, even jolly. Fun is the natural product of the human mind. It's so natural to be jolly that we are tempted to be so under the most serious circumstances. A lady once said to me that she daren't go to a funeral because everybody was so serious, and they made such hard work of it that she had to laugh aloud, and couldn't help it. It was funny to her to see how hard it is for people to be sorry and sad and wear serious faces, and it made her "bust right out," and she believed she'd explode at the funeral of her dearest friend. Her's may be a somewhat exceptional case, but we've all experienced something like this. It isn't natural to be unhappy, and fretting and worrying are miseries that were never intended to be a part and parcel of our living. They who entertain the opposite view, however, are numerous. They believe that the chief end of man is to stultify his soul and fret on forever. They are killing themselves, though, who believe this, and as I said in the start, more people kill themselves fretting than die of all other diseases and accidents combined. In fact, few die natural deaths, because they kill themselves worrying. Don't let's worry. Let's live out all our days. Let's have all the fun we can get out of life, and snap our fingers at what may be until it gets to us.

